# Serum IgG against Simian Virus 40 antigens are hampered by high levels of sHLA-G in patients affected by inflammatory neurological diseases, as multiple sclerosis

**DOI:** 10.1186/s12967-016-0981-y

**Published:** 2016-07-22

**Authors:** Roberta Rizzo, Silvia Pietrobon, Elisa Mazzoni, Daria Bortolotti, Fernanda Martini, Massimiliano Castellazzi, Ilaria Casetta, Enrico Fainardi, Dario Di Luca, Enrico Granieri, Mauro Tognon, The Emilia-Romagna network for Multiple Sclerosis (ERMES) study group

**Affiliations:** Section of Microbiology and Medical Genetics, Department of Medical Sciences, University of Ferrara, Via Luigi Borsari, 46, 44121 Ferrara, Italy; Section of Pathology, Oncology and Experimental Biology, Department of Morphology, Surgery and Experimental Medicine, University of Ferrara, Via Fossato di Mortara, 44121 Ferrara, Italy; Section of Neurology, School of Medicine, Department of Biomedical Sciences and Specialized Surgeries, University of Ferrara, Ferrara, Italy; Neuroradiology Unit, Department of Neurosciences, University Hospital of Ferrara, Ferrara, Italy

**Keywords:** SV40, HLA-G, Multiple sclerosis

## Abstract

**Background:**

Many investigators detected the simian polyomavirus SV40 footprints in human brain tumors and neurologic diseases and recently it has been indicated that SV40 seems to be associated with multiple sclerosis (MS) disease. Interestingly, SV40 interacts with human leukocyte antigen (HLA) class I molecules for cell entry. HLA class I antigens, in particular non-classical HLA-G molecules, characterized by an immune-regulatory function, are involved in MS disease, and the levels of these molecules are modified according with the disease status.

**Objective:**

We investigated in serum samples, from Italian patients affected by MS, other inflammatory diseases (OIND), non-inflammatory neurological diseases (NIND) and healthy subjects (HS), SV40-antibody and soluble sHLA-G and the association between SV40-prevalence and sHLA-G levels.

**Methods:**

ELISA tests were used for SV40-antibodies detection and sHLA-G quantitation in serum samples.

**Results:**

The presence of SV40 antibodies was observed in 6 % of patients affected by MS (N = 4/63), 10 % of OIND (N = 8/77) and 15 % of NIND (N = 9/59), which is suggestive of a lower prevalence in respect to HS (22 %, N = 18/83). MS patients are characterized by higher sHLA-G serum levels (13.9 ± 0.9 ng/ml; mean ± St. Error) in comparison with OIND (6.7 ± 0.8 ng/ml), NIND (2.9 ± 0.4 ng/ml) and HS (2.6 ± 0.7 ng/ml) subjects. Interestingly, we observed an inverse correlation between SV40 antibody prevalence and sHLA-G serum levels in MS patients.

**Conclusion:**

The data obtained showed a low prevalence of SV40 antibodies in MS patients. These results seems to be due to a generalized status of inability to counteract SV40 infection via antibody production. In particular, we hypothesize that SV40 immune-inhibitory direct effect and the presence of high levels of the immune-inhibitory HLA-G molecules could co-operate in impairing B lymphocyte activation towards SV40 specific peptides.

## Background

Multiple sclerosis (MS) is an inflammatory chronic demyelinating human disease of the central nervous system (CNS) characterized by an autoimmune pathogenic process in genetically predisposed individuals [1]. Infectious agents are environmental factors potentially involved in the MS onset. Distinct human viruses were found to be associated with MS, such as herpesviruses and retroviruses [2].

A recent study indicated that the simian polyomavirus SV40 seems to be associated with MS disease [3]. SV40, a neurotropic polyomavirus, is the causative viral agent of the progressive multifocal leukoencephalopathy (PML) in immune-compromised macaques. Many investigators detected SV40 footprints in human brain tumors and neurologic diseases [4]. SV40, as an adventitious virus, was present in early anti-polio vaccines administered to different human populations in the period 1955–1963. It is also possible that this polyomavirus was already present in humans, as unknown virus. Interestingly, SV40 interacts with human leukocyte antigen (HLA) class I molecules for cell entry [5]. After binding to class I molecules, SV40 enters cells via a unique endocytic pathway that involves caveolae and delivers SV40 to the endoplasmic reticulum. Although class I molecules bind SV40, they do not enter with SV40. Instead, they are shed from the cell surface by the activity of metalloproteases. HLA class I antigens, in particular non-classical HLA-G molecules, are involved in MS disease, and the levels of these molecules are modified according with the disease status [6–8]. HLA-G is characterized by the presence of membrane (m) bound (G1–G4) and soluble (s) (G5–G7) isoforms [6]. HLA-G is involved in mechanisms of immune tolerance in pregnancy, organ transplantation, autoimmune and inflammatory diseases by inhibiting cytolytic functions of natural killer cells, cytotoxic T lymphocytes, T and dendritic cells allo-proliferation [7]. Soluble and membrane-bound HLA-G isoforms have similar functions and interact with specific inhibitory receptors (ILT-2 and ILT-4) expressed by immune cells [9]. HLA-G has an important role in MS: (i) cerebrospinal fluid (CSF) levels and the intrathecal synthesis of sHLA-G are higher in MS patients in comparison with controls and are associated with clinical and radiological evidence of disease remission (ii) elevated sHLA-G concentrations in CSF correlated with the presence of an anti-inflammatory and pro-apoptotic intrathecal microenvironment, (iii) HLA-G expression is high within and around MS lesions and (iv) HLA-G + regulatory T cells highly represented in brain lesions of MS patients [8].

Interesting, during viral infections, HLA-G molecules are up-regulated by the virus as a mechanism of immune-escape [7] inhibiting the host immune response.

The recent development of specific and sensitive serologic test for SV40, that has allowed the detection of specific serum antibodies against SV40 VPs [1] and the possibility to evaluate the levels of soluble HLA-G expression in biological fluids [8], suggested the analysis of these two components in sera from MS patients.

The objective of the present study was to investigate whether serum samples from Italian patients affected by MS, other inflammatory diseases (OIND), non-inflammatory neurological diseases (NIND) and healthy subjects (HS) (i) carry SV40-antibodies, (ii) present different levels of soluble HLA-G molecules, (iii) the existence of an association between SV40-antobody prevalence and sHLA-G levels. The data obtained are relevant to understand the possible implication of SV40 infection and host immune response in MS disease.

## Methods

### Human samples

Serum samples (n = 282), collected at the University Hospital of Ferrara, Department of Neurology and Clinical Laboratory Analysis, were from our collections [3]. Human sera were from discarded clinical laboratory analysis samples, anonymously collected, coded with indications of age, gender and pathology, if any. Sera from MS patients (n = 63), mean age = 39 ± 26 years, as well as from other inflammatory neurologic dideases (OIND) (n = 77), mean age = 53 ± 10 years, and non-inflammatory neurologic diseases (NIND) (n = 59) affected patients, mean age = 54 ± 12 years, and healthy subjects (HS) (n = 83), mean age = 66 ± 5 years. Informed written consent was obtained by patients/individuals. The project was approved by the Ethics Committee, Ferrara, Italy.

### Synthetic peptides

Computer assisted analyses allowed the selection of two specific SV40 peptides, from the late viral region that did not cross-react with the BKV and JCV hyperimmune sera [1]. The two peptides belong to the VP1/VP2/VP3 viral capsid proteins (VP1 ID: 1489598; VP3 ID: 9486895; http://www.ncbi.nlm.nih.gov/nuccore). The amino acid sequences of the two peptides, known as VP1 B and VP2/3 C, respectively, are as follows:

VP1 B: NH2–NPDEHQKGLSKSLAAEKQFTDDSP–COOH

VP2/3 C: NH2–IQNDIPRLTSQELERRTQRYLRD–COOH

VP1 B and VP2/3 C mimotopes were selected as they react specifically in indirect ELISA with the rabbit hyperimmune serum that had been experimentally immunized with SV40 (positive control serum) and the amino acid residues of these two specific SV40 VP peptides show low homology with the BKV, JCV and other polyomaviruses VPs. SV40-positive and SV40-negative human sera were also employed as controls. These SV40 control sera, selected by the neutralization assay, were from our collections (see below the other technical details). BKV and JCV hyperimmune rabbit sera did not react with VP1 B or VP2/3 C peptides (negative control sera). The synthetic peptides were synthesized using standard procedures (UFPeptides s.r.l., Ferrara, Italy).

### Indirect enzyme-linked immunosorbent assay (ELISA)

Indirect ELISA with the mimotopes specific for the region of SV40 VPs were carried out as previously described [1]. We used as positive-control an immune rabbit serum containing anti-SV40 antibodies, as negative controls anti-BKV and anti-JCV immune sera, and three human serum samples, which were found to be SV40 negative in our previous investigations [1]. SV40-positive sera had a general cut-off, by spectrophotometric reading, in the range of 0.18 OD, that discriminates SV40-negative (OD < 0.18) from SV40-positive samples (OD > 0.18). As published before, in indirect ELISAs the human peptide hNPS [1] which is unrelated to SV40, was employed as a negative control peptide. This negative control peptide does not react with SV40-positive and SV40-negative sera, with OD values usually in the range of 0.088–0.098, similar to the OD background of both human and rabbit sera.

### SV40 specificity of the indirect ELISA employing synthetic peptides which mimic the VPs antigens

The identification and study of synthetic peptides was performed by comparative computer assisted analyses with BLAST program on the SV40 VP peptides VP1 B and VP2/3 C and the corresponding amino acid (a.a.) sequences of the new human polyomaviruses (HPyV) and hundreds of different BKV and JCV serotypes. Results indicate a low homology for the BKV and JCV prototypes and other polyomaviruses [1].

### sHLA-G detection by ELISA

sHLA-G concentrations were investigated in serum samples by enzyme immunosorbent assay, as reported in the Essen Workshop on sHLA-G quantification [10]. As capture antibody we used MEM-G9 MoAb (Exbio, Praha, CZ) and anti-beta2 microglobulin MoAb—HRP was used as detection antibody. The intra-assay coefficient of variation (CV) is 1.4 %, the inter-assay CV is 4.0 %. The limit of sensitivity is 1.0 ng/ml.

### Statistical analyses

All analyses were performed by Prism 4.0 (GraphPad software). To determine significances we used two-sided Chi square test, Anova and Newman-Keuls comparison test, nonparametric Spearman analysis and Fisher’s exact test. For all tests, we considered p < 0.05 to be statistically significant.

## Results

**Fig. 1:**
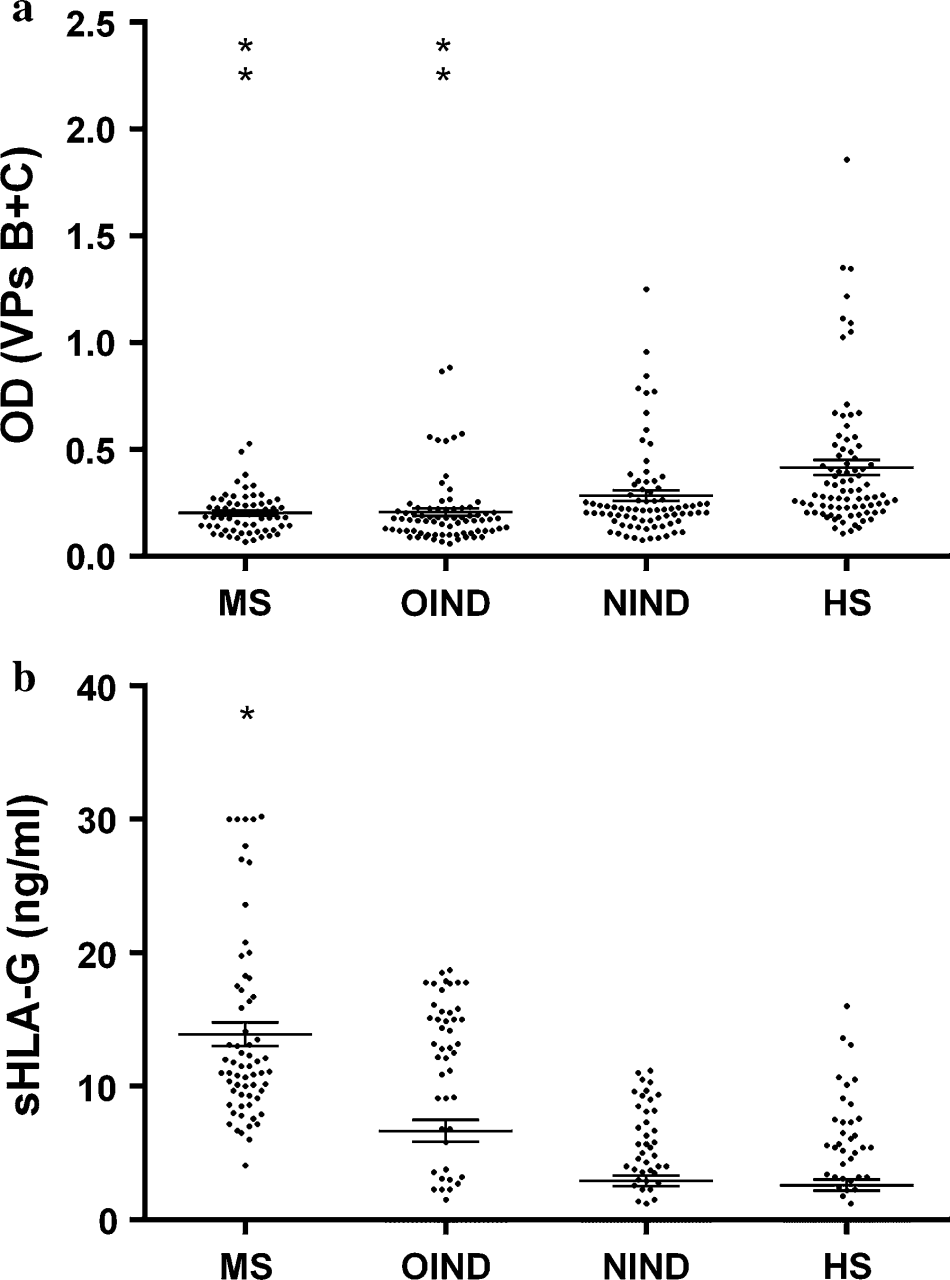
SV40 antibodies titres and sHLA-G levels in serum samples from from *MS* multiple sclerosis patients , *OIND* other inflammatory neurologic diseases , *NIND* non-inflammatory neurologic diseases and *HS* Healthy subjects. **a** SV40 antibodies titres are are presented as values of *OD* optical density readings at λ 405 nm of serum samples diluted at 1:20, detected in indirect ELISA. In *scatter dot* plotting, each plot represents the dispersion of OD values to a mean level indicated by the *line* inside the scatter with Standard Error Mean (SEM) for each group of subjects analyzed. **b** sHLA-G levels are presented as ng/ml in serum samples diluted at 1:2, detected in indirect ELISA. In *scatter dot* plotting, each plot represents the dispersion of sHLA-G values to a mean level indicated by the *line* inside the scatter with Standard Error Mean (SEM) for each group of subjects analyzed. Statistical analysis was performed using Anova and Newman-Keuls comparison test. (**p < 0.0001; *p < 0.001)

We evaluated SV40 antibodies seroprevalence and sHLA-G serum levels in 4 cohorts of subjects: MS, OIND, NIND and HS subjects, matched for sex and age. Two indirect ELISAs, against two distinct SV40 viral capsid protein (VP) epitopes, named B and C, were employed to test serum samples. The two indirect ELISAs gave overlapping results. Serum samples, which reacted with both mimotopes, and considered SV40-positive, reached an overall prevalence of 6 % (4/63) in MS, 10 % (8/77) in OIND, 15 % (9/59) in NIND and 22 % (18/83) in HS (Table 1). It is interesting to note that the percentage of seroprevalence in MS patients is much lower than that of HS subjects (p < 0.05; Fisher’s exact test) [3]. Among sera of the different cohorts, MS, OIND, NIND, HS, 64 samples tested positive for the epitope B with a prevalence of 23 %, whereas 54 samples tested positive for the epitope C with a prevalence of 19 %. The difference is not statistically significant (p < 0.05, by Fisher’s exact test). The mean OD of sera (VPs B + C ± Std Error) in MS (0.20 ± 0.01) and OIND (0.21 ± 0.02) were lower than that in HS (0.41 ± 0.03) and in NIND (0.35 ± 0.024) subjects (Anova and Newman-Keuls comparison test; p < 0.0001) (Table 1; Fig. 1a).

**Table 1:** SV40 and sHLA-G results in MS, OIND, NIND and HS subjects

	SV40 Ab titre OD (mean ± Std error)^a^	SV40 Ab + (%)^b^	SHLA-G ng/ml (mean ± Std error)^a^	SHLA-G + (%)^b^	<Cut off sHLA-G (> 15 ng/ml)^b^
MS (63)	0.20 ± 0.01**	6*	13.9 ± 0.9 ng/ml**	100**	30***
OIND (77)	0.21 ± 0.02**	10*	6.7 ± 0.8 ng/ml	58	29***
NIND (59)	0.35 ± 0.024	15	2.9 ± 0.4 ng/ml	51	0
HS (83)	0.41 ± 0.03	22	2.6 ± 0.7 ng/ml	41	2

p values: * p < 0.05; ** p ≪ 0.0001; *** p <0.001

^a^Anova and Newman-Keuls comparison test

^b^Fisher exact test

Then, the same serum samples were analyzed by ELISA for the presence of sHLA-G molecules. All MS serum samples were positive for sHLA-G (63/63: 100 %), whereas sHLA-G molecules were detected in 58 % (45/77) of OIND, 51 % (20/59) of NIND and 41 % (35/83) of HS (p < 0.0001; Fisher’s exact test) (Table 1). When we looked at sHLA-G expression, we observed higher mean sHLA-G levels in sera of MS (13.9 ± 0.9 ng/ml) and OIND (6.7 ± 0.8 ng/ml) patients in comparison with NIND (2.9 ± 0.4 ng/ml) and HS (2.6 ± 0.7 ng/ml) subjects (Anova and Newman-Keuls comparison test; p < 0.001) (Table 1; Fig 1b). These results are in agreement with our previous data on higher sHLA-G levels in MS patients in comparison with control cohorts [7].

When we evaluated the 95 %CI of mean for choosing a cutoff point for sHLA-G levels that differentiates MS patients in comparison with control cohorts, we obtained the value of 15 ng/ml. We observed that MS patients presented levels of sHLA-G higher than the cutoff value in the 30 % (19/63) of samples, OIND in the 29 % (13/45), NIND in the 0 % (0/20) and HS in the 3 % (1/35). Of note, MS patients positive for the presence of antibodies reacting with SV40 mimotopes presented sHLA-G levels lower than 15 ng/ml, revealing an inverted correlation between the prevalence of antibodies reacting with SV40 mimotopes and the sHLA-G levels. (Nonparametric correlation Spearman analysis; r = 0.997, p = 0.0001).

On the contrary, no correlation between the presence of antibodies reacting with SV40 mimotopes and the sHLA-G levels was observed in control cohorts.

The originality of the findings reported herein is the inverted association between SV40 antibody prevalence and sHLA-G concentration detected in sera of MS patients.

## Discussion

In this study, serum samples from patients affected by neurologic diseases, including MS, were analyzed for exposure to SV40 infection, as reported before [3]. In this investigation, serum samples were analyzed for the first time by ELISA to verify the sHLA-G levels. At the same time, samples belonging to the serum collection reported in a previous study [3] were re-analyzed by ELISA with SV40 synthetic peptides B and C for the viral antibody prevalence. The SV40 antibody prevalence detected herein did not differ statistically from early data [3].

Our immunologic data suggest that specific SV40 antibodies are detectable in human serum samples from neurologic patients and healthy individuals. Specifically, the presence of SV40 antibodies observed in patients affected by MS (6 %) revealed a lower prevalence of SV40 antibodies in respect to controls, HS (22 %). It should be noted that MS patients included in our study, at the time of the serum collection, were not subjected to any immuno-modulatory therapy and were in a remission phase of the disease. These data indicate that the low prevalence of SV40 antibodies is not due to the immune-modulatory therapy or to a status of reactivation of the disease. We hypothesize that patients affected by inflammatory neurologic diseases, including MS, are unable to counteract SV40 infection via antibody production.

Our results on soluble HLA-G levels molecules support our previous data on HLA-G expression in MS patients [7]. In particular, MS patients are characterized by higher sHLA-G serum levels in comparison with OIND, NIND and healthy subjects. The increased expression of HLA-G secretion in MS and OIND patients could account for the inflammatory condition, that these patients try to counteract at the systemic level, via the production of an anti-inflammatory molecule.

The novelty of this research is the impressive inverse correlation between sHLA-G serum levels and the prevalence of SV40 antibodies in MS patients. In particular, we observed that MS patients positive for SV40 antibodies presented sHLA-G levels lower than 15 ng/ml, the cutoff point for sHLA-G levels that differentiate MS patients in comparison with NIND and HS cohorts. OIND patients behave similarly to MS patients, with a lower prevalence of antibodies reacting with SV40 mimotopes and >15 ng/ml sHLA-G levels. In the other two cohorts, represented by NIND patients and HS, a higher prevalence of SV40 antibodies and <15 ng/ml sHLA-G levels were revealed. These results suggest that the presence of >15 ng/ml levels of sHLA-G seems to hamper the production of SV40 antibodies, in a manner that remains to be elucidated. In this context, it should be recalled that the engagement of the surface Ig-like transcript 2 (ILT2) inhibitory receptor with its preferential ligand HLA-G is critical to inhibit B cell functions [9]. Indeed, ILT2-HLA-G interaction impedes both naive and memory B cell functions in vitro and in vivo. Particularly, HLA-G inhibits B cell proliferation, differentiation, and Ig secretion in both T cell-dependent and -independent models of B cell activation. Interestingly, SV40 down-regulates the expression of CD83 and CD86 on dendritic cells (DC) and impairs DC-induced activation of T cell and B cell proliferations [11]. These findings suggest that SV40 infection might also cause immune suppression by influencing differentiation and maturation of B cells. We may speculate that the low prevalence of SV40 specific antibodies in MS patients could be in part ascribed to SV40 immune-inhibitory direct effect and in part to the presence of >15 ng/ml HLA-G levels that could impair B lymphocyte activation towards SV40 specific peptides. Since both MS disease and viral infection are characterized by an increase in HLA-G expression [7, 8] we could hypothesize a synergic effect of these two conditions in the maintenance of a dysregulated immune response in MS patients. To account this hypothesis, we will evaluated the same variable in larger cohorts of subjects, correlating the results with clinical follow-up.

## Conclusions

Our data confirm a low prevalence of SV40 antibodies in MS patients (3). These results seem to be due to a generalized status of inability to counteract SV40 infection via antibody production. In particular, we hypothesize that SV40 immune-inhibitory direct effect and the presence of high levels of the immune-inhibitory HLA-G molecules could co-operate in impairing B lymphocyte activation towards SV40 specific peptides. Our findings are of extreme interest to understand the possible role of SV40 infection in the pathogenesis of MS disease and the effect of this virus on the host immune response.
